# Comparative study on clinical outcomes in autologous chondrocyte implantation using three-dimensional cultured JACC^®^ with collagen versus periosteum coverings

**DOI:** 10.1038/s41598-024-59798-7

**Published:** 2024-04-29

**Authors:** Yuki Kato, Shin Yamada, Shuzo Takazawa, Soichi Hattori, Takuya Okada, Hiroshi Ohuchi

**Affiliations:** Department of Sports Medicine, Kameda Medical Center, 929 Higashi-Cho, Kamogawa City, Chiba Prefecture 296-8602 Japan

**Keywords:** Medical research, Trauma

## Abstract

This study investigates the efficacy of a collagen membrane as a substitute for autologous periosteum in atelocollagen-assisted autologous chondrocyte implantation (ACI) using J-TEC autologous cultured cartilage (JACC®). Sixty-nine patients with knee joint chondral defects underwent ACI using JACC®—34 with periosteum-covered ACI (P-ACIs) and 35 with collagen-covered ACI (C-ACIs). Clinical outcomes were compared through patient-reported measures, International Cartilage Repair Society (ICRS) Cartilage Repair Assessment (CRA) scores at second-look arthroscopy one year postoperatively, and adverse event incidence. Postoperative subjective scores significantly improved up to two years, with no significant differences between P-ACI and C-ACI groups. However, C-ACI exhibited a lower adverse event rate (p = 0.034) and significantly higher ICRS CRA scores (p = 0.0001). Notably, C-ACI outperformed P-ACI in both femoral condyle and trochlea assessments (p = 0.0157 and 0.0005, respectively). While clinical outcomes were comparable, the use of a collagen membrane demonstrated superiority in ICRS CRA during second-look arthroscopy and adverse event occurrence.

## Introduction

Articular cartilage injuries rarely heal naturally because of the lack of nerves, blood vessels, and lymphatic vessels^1^. Localized cartilage defects are treated by bone marrow stimulation or osteochondral autograft transplantation^2^. For large chondral defects that are difficult to repair with these treatments, Brittberg et al.^3^ introduced autologous chondrocyte implantation (ACI) in 1994. This technique involves the injection of chondrocyte suspension into the injured cartilage, followed by covering it with an autologous periosteal patch, and is reported to have good clinical results^4,5^. However, there are some disadvantages, such as heterogeneity in the quality of the autologous periosteum, complexity, and invasiveness of the transplantation procedure, which mainly consists of collecting the autologous periosteum from the proximal tibia and suturing the periosteal patch to the transplant site, and a high incidence of post-transplantation complications, including periosteal hypertrophy^6–8^. Therefore, methods for solving these problems are continuously being developed^9^. Recently, using porcine collagen membranes as patches instead of autologous periosteum has become a mainstream technique, especially in Europe and the United States^10^.

In Japan, J-TEC autologous cultured cartilage (JACC®, Japan Tissue Engineering Co., Ltd., Aichi Prefecture) has been covered by National Health Insurance since April 2013. JACC® was made to order for each patient by isolating chondrocytes from healthy cartilage tissue collected from the patient and embedding them in an atelocollagen gel for culture. In the early JACC® implantation method, cultured cartilage was transplanted into the defect and patched with the periosteum harvested from the proximal tibia. There have been several reports of JACC® implantation using periosteal patches, all of which have reported excellent results^11–13^. Adachi et al.^14^ evaluated the International Cartilage Repair Society (ICRS) Cartilage Repair Assessment Score (CRA) (ICRS Cartilage Injury Evaluation Package (http://www.cartilage.org/) two years after JACC® implantation using a periosteal patch and reported excellent results. However, in response to some of the problems caused by using the autologous periosteal patch, as described above, the use of a collagen membrane (Chondro-Gide®, Geistlich Pharma AG, Wolhusen, Switzerland) for JACC® implantation was approved in January 2019.

Several reports have compared the use of periosteum-covered ACI (P-ACI) with collagen-covered ACI (C-ACI), and collagen membranes have provided better results^6–8,15–17^. Most of these studies showed the effectiveness of collagen membranes in terms of survival rate and incidence of adverse events, but few reports have used ICRS CRA^6^ for evaluation. Gooding et al.^6^ compared P-ACI and C-ACI using the ICRS CRA assessment based on arthroscopic findings and reported no significant difference between the two groups. However, the ACI procedure used in this study was the first generation and involved injecting a chondrocyte suspension. However, no reports compare P-ACI and C-ACI with JACC® implantation, a next-generation ACI involving three-dimensional cultured cartilage, using ICRS CRA. Furthermore, there are no reports on the clinical score or degree of cartilage repair after JACC® implantation using a collagen membrane.

The purpose of this study was to report the postoperative clinical score and degree of cartilage repair in JACC® implantation cases and to compare the results between P-ACI and C-ACI. We hypothesized that using collagen membranes would improve the outcomes of JACC® implantation.

## Methods

Ethical approval for this study was obtained from the ethics committee of Kameda Medical Center (approval No. 21-135). Informed consent was obtained in the form of opt-out on the web-site. Those who rejected were excluded. This study was conducted retrospectively. This study was conducted in accordance with the Declaration of Helsinki. The study involves autologous cultured cartilage transplantation using the patients' own tissue. This means that no tissues were provided by third parties such as prisoners. From January 2017 to December 2020, 69 patients (31 males and 38 females) who underwent ACI for the knee joint's chondral defects were included in this study. The ACI performed in this study was atelocollagen-assisted autologous cartilage implantation using JACC®. The indications for ACI were large chondral defects (grade three or four) of at least 4 cm^2^ and trauma or osteochondral dissecans as the etiology of cartilage defects. The subjects were patients who underwent second-look arthroscopy one year after ACI. Patients with arthritis due to collagen diseases such as rheumatoid arthritis were excluded.

The patients' backgrounds are presented in Table 1. The mean age at surgery of the subjects (n = 69) was 47.8 ± 1.1 years (16–66 years). The mean body mass index was 24.5 ± 0.4. The preoperative cartilage defect range averaged 5.79 ± 0.43 cm^2^ (0.61 to 17.65). Twenty knees had single lesions, and 49 knees had multiple lesions. A total of 108 cartilage defects were observed. The average follow-up period was 96 weeks (52.4–120.4 weeks).

**Table 1:** Patients background.

Patient and defect characteristics	(Number of subjects with available data)			
	Total(69)	P-ACI (34)	C-ACI (35)	
Mean age (years)	47.8 (69)	49.1 (34)	46.7 (35)	P = 0.2733 (student's t-test)
Mean body mass index (kg/m^2^)	24.5 (69)	24.5 (34)	24.5 (35)	P = 0.9969 (student's t-test)
Gender				
Male	45% (31)	41% (14)	49% (17)	P = 0.6307 (Fisher's exact test)
Female	55% (38)	59% (20)	51% (18)	
Disease				
Trauma	97% (67)	97% (33)	97% (34)	P = 1.0000 (Fisher's exact test)
Osteochondritis dissecans	3% (2)	3% (1)	3% (1)	
Mean lesion size (cm^2^/knee)	**5.79 (69)**	**5.84 (34)**	**5.75 (35)**	P = 0.9188 (student's t-test)
Single lesion	29% (20)	26% (9)	31% (11)	P = 0.7918 (Fisher's exact test)
Multiple lesion	71% (49)	74% (25)	69% (24)	
Defect location	**108**	**62**	**46**	
Medial femoral condyle	43% (30)	53% (18)	34% (12)	
Lateral femoral condyle	32% (22)	44% (15)	20% (7)	
Medial Tibial plateau	4% (3)	9% (3)	0% (0)	
Lateral Tibial plateau	7% (5)	12% (4)	3% (1)	
Patella	9% (6)	9% (3)	9% (3)	
Trochlea	61% (42)	56% (19)	66% (23)	
Concomitant surgical procedures				
Around knee osteotomy	52% (36)	50% (17)	54% (19)	
*High tibial osteotomy*	33% (23)	35% (12)	31% (11)	
*Distal femoral osteotomy*	17% (12)	12% (4)	23% (8)	
*Double level osteotomy*	1% (1)	3% (1)	0% (0)	
Other cartilage repairs combined with ACI	77% (53)	74% (25)	80% (28)	
*Microfracture*	29% (20)	18% (6)	40% (14)	
Osteochondral autograft *Transplantation*	72% (50)	68% (23)	77% (27)	
Meniscus repair	23% (16)	29% (10)	17% (6)	
Cruciate ligament reconstruction	10% (7)	9% (3)	11% (4)	
Lateral retinacular release	22% (15)	0% (0)	43% (15)	

P-ACI, periosteum-covered atelocollagen-assisted autologous cartilage implantation; C-ACI, collagen-covered atelocollagen-assisted autologous cartilage implantation.

From January 2017 to February 2019, the P-ACI group consisted of 34 patients (14 males and 20 females) with an average age of 49.1 ± 1.6 years (19–66 years). In Japan, the use of type I/III collagen membranes instead of autologous periosteum began in 2019. From March 2019 to February 2020, the C-ACI group consisted of 35 patients (17 males and 18 females) with an average age of 46.7 ± 1.6 years (16–56 years). After the introduction of the collagen membrane, the use of periosteum membrane has been discontinued. Therefore, the two interventions were not allocated based on randomization.

### Surgical technique

JACC® was approved by Japan's Pharmaceuticals and Medical Devices Agency and manufactured by Japan Tissue Engineering Co., Ltd. (J-TEC, Aichi, Japan). Approximately 0.4 g of cartilage tissue was arthroscopically harvested from non-weight-bearing areas of the patient's knee and sent to J-TEC. Processing of cartilage tissue was conducted at a cell culture processing facility in compliance with the ministerial ordinance that outlines regulatory requirements for manufacturing regenerative medical and related products, referred to as 'Good Gene, Cellular, and Tissue-based Products Manufacturing Practice (GCTP),' as specified by the Ministry of Health, Labour and Welfare. Cartilage pieces were briefly subjected to enzymatic digestion, and isolated chondrocytes were embedded in an atelocollagen gel and cultured for four weeks in a three-dimensional manner. After rigorous shipping inspection, JACC® was sent to our hospital under temperature control for surgery.

**Figure 1:**
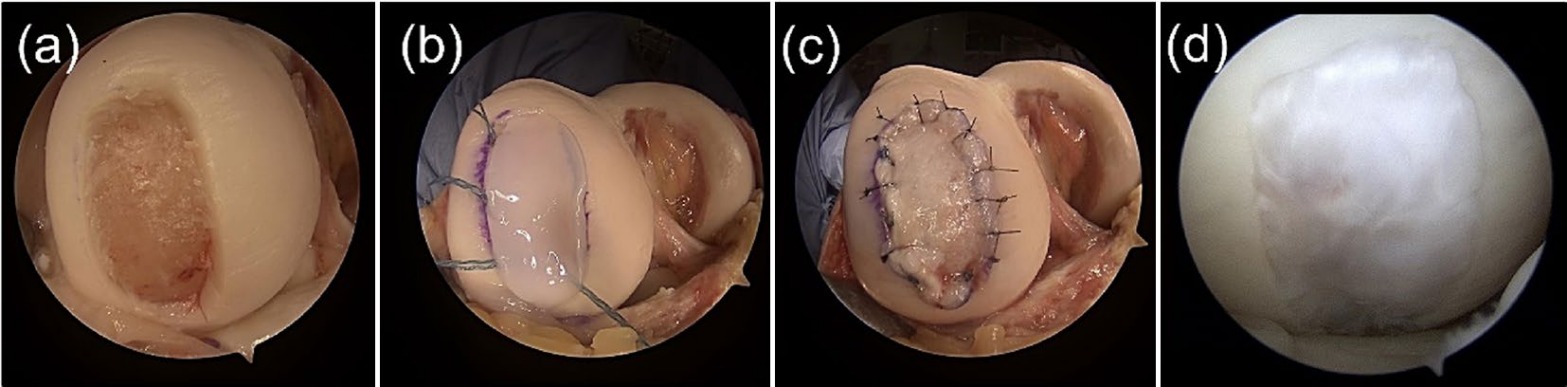
Periosteum covered ACI and its second-look arthroscopy findings. (**a**) chondral defect site. (**b**) implanted cultured cartilage sheet. (**c**) a patch of periosteum, (**d**) second-look arthroscopy findings one year after the surgery. ACI, autologous chondrocyte implantation.

**Figure 2:**
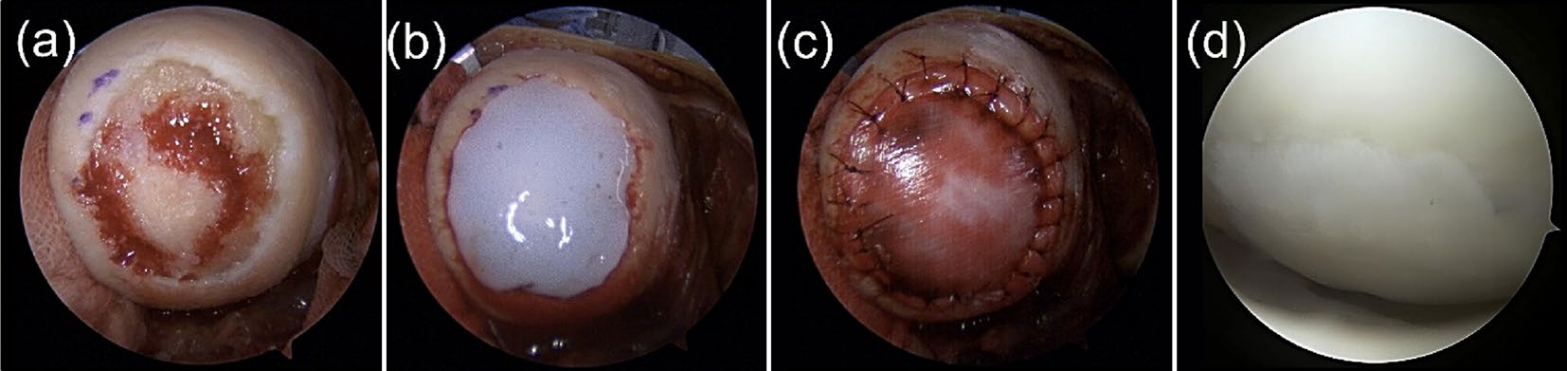
Collagen covered ACI and its second-look arthroscopy findings. (**a**) chondral defect site. (**b**) implanted cultured cartilage sheet. (**c**) a patch of collagen membrane, (**d**) second-look arthroscopy findings one year after the surgery. ACI, autologous chondrocyte implantation.

All the surgeries were performed by a single surgeon (Y. K.). During implantation surgery, the cartilage defect site was exposed by knee joint arthrotomy, and the cartilage defect lesion was curetted to remove the fibrous tissue and calcified layer. The irregular unevenness of the subchondral bone was flattened with a surgical bur. The floating articular cartilage around the cartilage defect was also excised so that the implanted cultured cartilage was in direct contact with normal articular cartilage. A three-dimensional cultured cartilage sheet (JACC®) was placed on the defect and covered with a patch of the periosteum (Fig. 1) or a collagen membrane (Chondro-Gide®, Geistlich Pharma AG, Wolhusen, Switzerland) (Fig. 2). The patch was sutured to the surrounding normal cartilage with a 6–0 nylon thread or absorbent thread at 2 mm intervals. At the same time, concomitant surgical procedures were performed, as described in Table 1.

### Rehabilitation

In each case, the site and size of the articular cartilage lesion were different, and the biological repair process of the cartilage tissue and biomechanical characteristics of the knee joint were also different. A postoperative step-by-step rehabilitation program was developed for each patient. Mechanical loading of articular cartilage promotes extracellular matrix synthesis and turnover^18,19^. Mechanically unloaded environments such as non-weight bearing and immobilization result in biologically adverse changes such as nutritional deficiencies, decreased cartilage hardness, and thickness, and decreased proteoglycans^20,21^. However, since the cartilage transplantation site in the early postoperative period was structurally fragile, it was not loaded until eight to 12 weeks after the operation when the transplanted cartilage sheet was considered to adhere to the subchondral bone. Although a weight-bearing gait in the extension position is possible from a relatively early stage following ACI for patellofemoral joints, the knee brace prevents excessive shearing force to the transplant site for up to three months after surgery. From 12 weeks after surgery, mechanical stress exercises targeting the transplant site were gradually permitted. Training such as jogging for resuming sports activities could be started nine months after the operation. One year after the procedure, return to sports was permitted depending on the recovery of muscle strength.

### Outcome measures

All patients were assessed preoperatively with the following subjective outcome scores: International Knee Documentation Committee (IKDC) score, Lysholm score, and Knee Injury and Osteoarthritis Outcome Score (KOOS). They were also evaluated at three, six, nine, 12, 18, and 24 months postoperatively using the same clinical evaluations. A second-look assessment was performed one year postoperatively. The ACI site was assessed following the ICRS CRA to obtain data on the quality of the regenerated cartilage and adverse events.

### Statistical analysis

A Student's t-test was performed on mean age, body mass index, and cartilage defect size to compare patient background between the two groups. Fisher's exact test was performed for sex differences, disease (trauma or osteochondral dissecans), and the presence of multiple lesions. Student's or Welch's t-test was performed to compare the two groups at each time point for each clinical score. Student's or Welch's t-test was also performed to compare the preoperative and postoperative scores (at three, six, nine, 12, 18, and 24 months). The Wilcoxon rank-sum test was performed to compare the two groups for the ICRS CRA score one year after surgery. Wilcoxon rank-sum test was also performed for the ICRS CRA score at each transplant site. Using the Holm procedure, the P-values for the IKDC score, Lysholm score, and KOOS were adjusted for comparisons at multiple time periods. The P-values for other endpoints were not adjusted for multiplicity. Statistical significance was set at P < 0.05. Statistical analysis was performed using the SAS statistical software (SAS Institute Japan Ltd., version 9.4).

## Results

### Description of study population

Table 1 describes the study population. The mean follow-up period was 104 weeks in the P-ACI group and 88.3 weeks in the C-ACI group. The P-ACI group had a longer follow-up period than the C-ACI group. Since collagen membranes were later used instead of periosteal, the two groups had significant different follow-up period. There were no statistically significant differences between the two groups in terms of mean age (p = 0.2733), body mass index (p = 0.9969), sex differences (p = 0.6307), disease (traumatic or OCD) (p = 1.0000), cartilage defect size (p = 0.9188), or the presence of multiple lesions (p = 0.7918).

### Clinical evaluation

**Figure 3:**
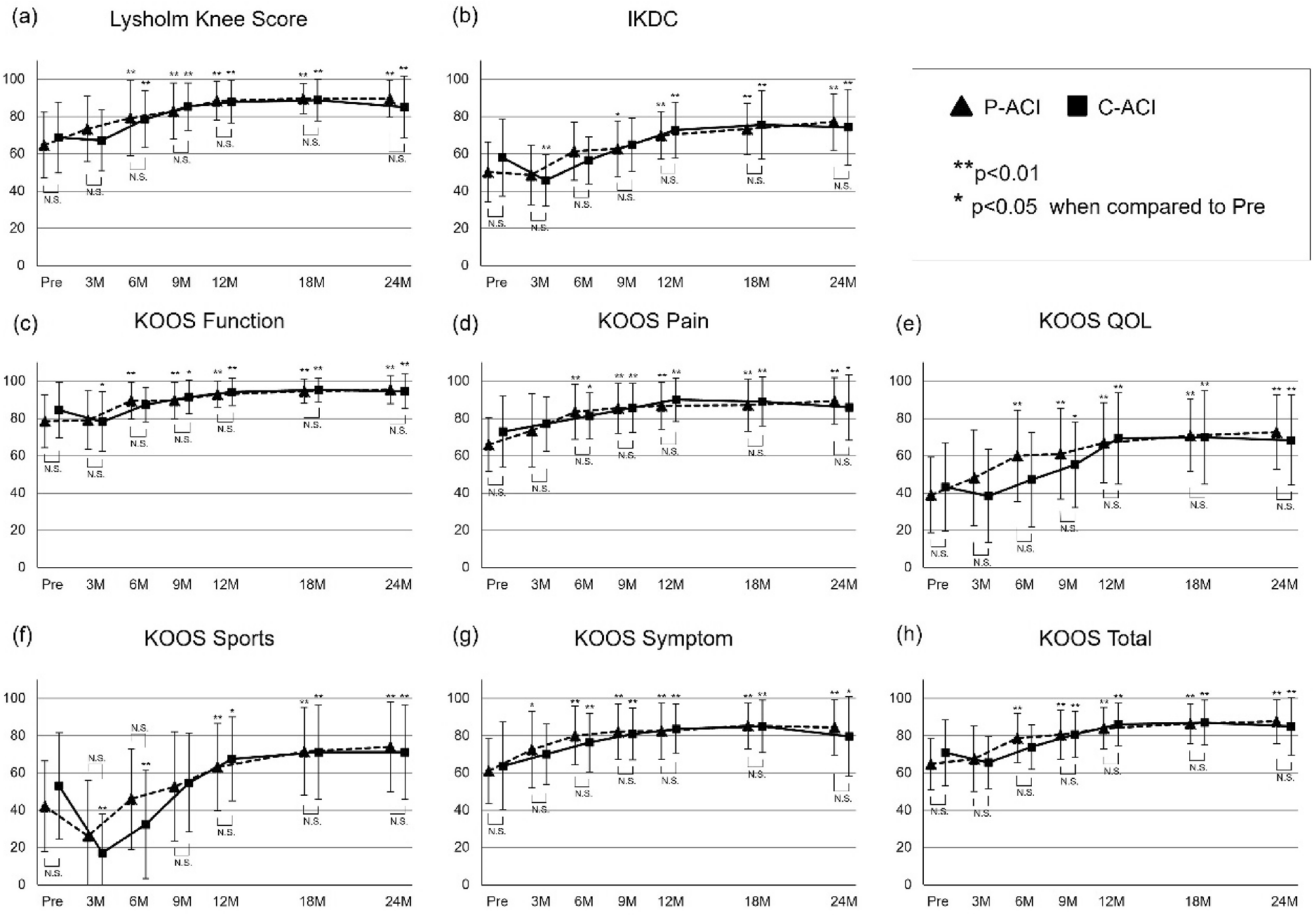
Clinical subjective scores. (**a**) Lysholm knee score, (**b**) IKDC, (**c**) KOOS function, (**d**) KOOS pain, (**e**) KOOS QOL, (**f**) KOOS sports, (**g**) KOOS symptom, and (**h**) KOOS total. P-values were adjusted for multiplicity with the use of the Holm procedure. IKDC, International Knee Documentation Committee; KOOS; Knee Injury and Osteoarthritis Outcome Score.

Regardless of the difference in covering membrane, all clinical subjective scores, including Lysholm knee score (Fig. 3a), IKDC (Fig. 3b), and KOOS (Fig. 3c–h), were significantly improved when comparing the postoperative two years to the preoperative scores. There was no significant difference in the scores of the P-ACI and C-ACI groups for each postoperative score up to two years after surgery (Fig. 3). No significant difference was observed between the P-ACI and C-ACI groups in the amount of change from preoperative scores to postoperative scores up to two years after surgery.

### Adverse events

The incidence of adverse events is shown in Table 2. The number of cases in "Total" is not the total number of adverse events because one case with two or more adverse events is counted as one case. The incidence of adverse events was 18.8% in 13 out of 69 knees, 29.4% in 10 of 34 knees of the P-ACI group, and 8.6% in three of 35 knees in the C-ACI group. The incidence of adverse events, regardless of the type, was significantly higher in the P-ACI group than in the C-ACI group (p = 0.034).

**Table 2:** Incidence of adverse events.

Adverse events	Total (69)	P-ACI (34)	C-ACI (35)	Fisher's exact test
Total	19% (13)	29% (10)	9% (3)	p = 0.0340
Delamination	12% (8)	15% (5)	9% (3)	p = 0.4773
Hypertrophy	0% (0)	0% (0)	0% (0)	
Ossification	0% (0)	0% (0)	0% (0)	
Hydrarthrosis	4% (3)	6% (2)	3% (1)	p = 0.6139
Knee contracture	6% (4)	12% (4)	0% (0)	p = 0.0536

P-ACI, periosteum-covered atelocollagen-assisted autologous cartilage implantation; C-ACI, collagen-covered atelocollagen-assisted autologous cartilage implantation.

The total incidence of delamination was 12%. The P-ACI group tended to have a higher incidence rate, with 15% for P-ACI and 9% for C-ACI. Neither hypertrophy nor ossification was observed in either group. There was no statistically significant difference in the incidence of each adverse event.

### ICRS CRA on second-look arthroscopy

Table 3 shows the ICRS CRA grade on second-look arthroscopy for the P-ACI group (34 knees, 62 sites) and C-ACI group (35 knees, 46 sites) one year after surgery. In the P-ACI group, 22.6%, 41.9%, 21.0%, and 14.5% of the cases were grades I, II, III, and IV, respectively. In the C-ACI group, 65.2%, 26.1%, 2.2%, and 6.5% of the cases were grades I, II, III, and IV, respectively.

**Table 3:** The ICRS CRA grade on second-look arthroscopy

	P-ACI (N = 62)					C-ACI (N = 46)				
**ICRS CRA grade**	**Total**	FC	Tibia	Trochlea	Patella	**Total**	FC	Tibia	Trochlea	Patella
**I. Excellent**	**14 (22.6%)**	7	0	5	2	**30 (65.2%)**	11	0	18	1
**II. Good**	**26 (41.9%)**	14	1	10	1	**12 (26.1%)**	6	1	4	1
**III. Fair**	**13 (21.0%)**	7	3	3	0	**1 (2.2%)**	1	0	0	0
**IV. Poor**	**9 (14.5%)**	5	3	1	0	**3 (6.5%)**	1	0	1	1

ICRS CRA, International Cartilage Repair Society Cartilage Repair Assessment Score; P-ACI, periosteum-covered atelocollagen-assisted autologous cartilage implantation; C-ACI, collagen-covered atelocollagen-assisted autologous cartilage implantation.

### Comparison of both groups in ICRS CRA score

**Figure 4:**
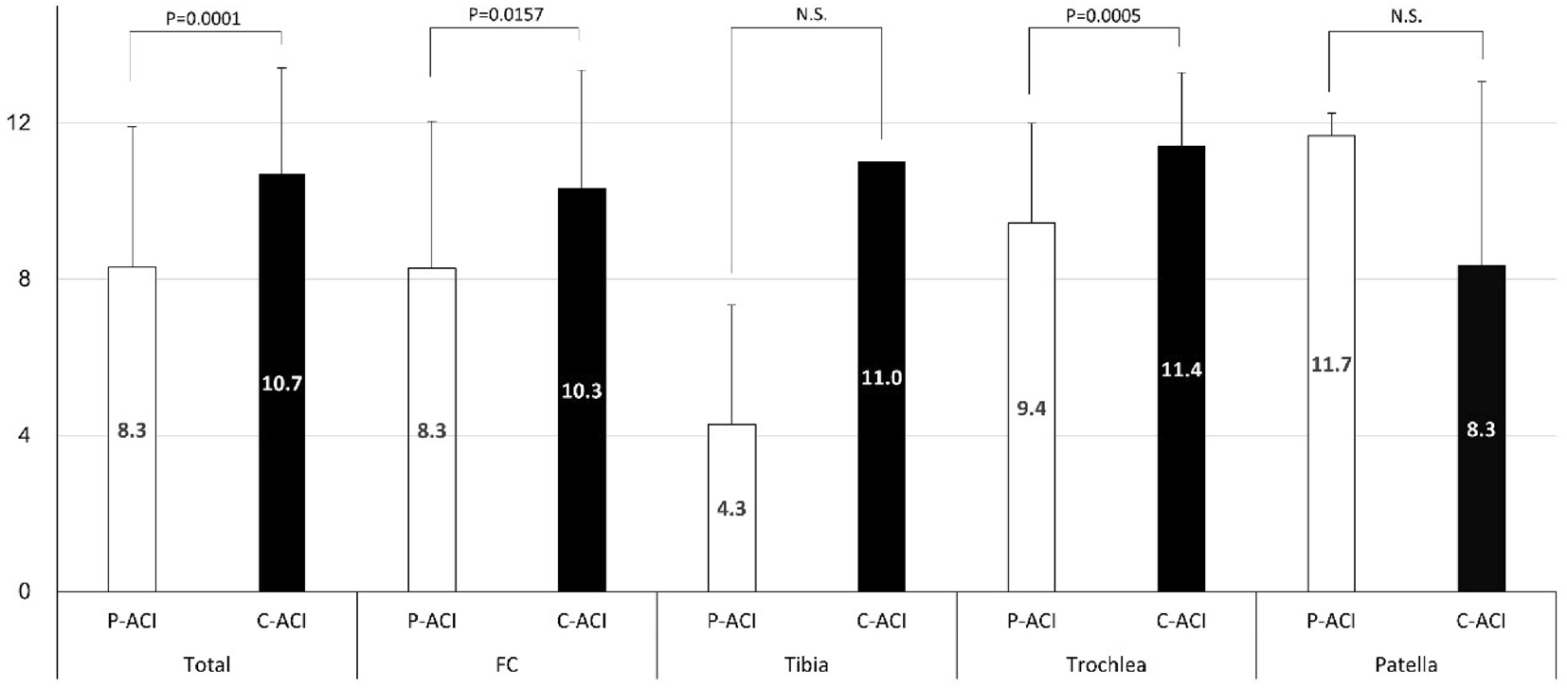
Comparison of both groups in ICRS CRA score. ICRS CRA, International Cartilage Repair Society Cartilage Repair Assessment Score; P-ACI, periosteum-covered atelocollagen-assisted autologous cartilage implantation; C-ACI, collagen-covered atelocollagen-assisted autologous cartilage implantation; FC, femoral condyle.

The label 'total' compares the ICRS CRA scores between both groups regardless of the transplant site and also compares the ICRS CRA scores for both groups at each transplant site (femoral condyle, tibia, trochlea, and patella) (Fig. 4). The average ICRS CRA score was significantly higher in the C-ACI group than in the P-ACI group, irrespective of the implantation site (p = 0.0001). The ICRS CRA score was significantly higher in the CACI group than in the P-ACI group for both the femoral condyle and trochlea (p = 0.0157 and 0.0005, respectively). There was no statistically significant difference between the two groups in the patellar ICRS CRA score. The small sample size of the tibial plateau precluded statistical testing between the two groups.

## Discussion

This study is the first to report the clinical results of C-ACI using JACC®, the only ACI product approved by the Pharmaceuticals and Medical Devices Agency in Japan. Adachi et al.^14^ have reported the clinical results of P-ACI using the JACC®. Instead of the periosteum, collagen membranes have been available in Japan since 2019. However, the clinical results of JACC® transplantation with a collagen membrane cover have not been reported.

In this study, the clinical results of ACI using JACC® were compared between C-ACI and P-ACI. Gooding et al.^6^ compared collagen-covered and periosteum-covered first-generation ACI, in which a cell suspension was injected. The first-generation ACI had problems such as leakage of chondrocytes from the transplanted site, uneven distribution of chondrocytes, and decreased matrix productivity due to the monolayer culture. JACC®, a three-dimensional cultured cartilage with atelocollagen as a scaffold, can alleviate these problems.

Matrix-induced autologous chondrocyte implantation (MACI®), approved by the Food and Drug Administration, is also a three-dimensional culture ACI that uses a collagen membrane as a scaffold. As a follow-up to Zeifang's study^22^, Barié et al.^23^ reported long-term results comparing MACI® and P-ACI (1st generation). Their study could not make a simple comparison between collagen and periosteal coverings because they compared monolayer-cultured ACI (first generation) and three-dimensional cultured ACI without collagen covering (third generation). We compared the periosteum and collagen membrane in atelocollagen-assisted three-dimensional cultured ACI because JACC® was used in all cases in the present study.

This study showed that C-ACI was significantly superior to P-ACI in the standard ICRS CRA on second-look arthroscopy one year after surgery. Gooding et al.^6^ previously compared ICRS CRA between C-ACI and P-ACI. They showed no significant difference in the ICRS CRA or the superiority of collagen membranes in both groups. In their report^6^, the percentage of each ICRS grade in P-ACI and C-ACI one year after surgery were 10.3% and 9.7% for grade I, 69% and 71% for grade II, 17.2% and 16% for grade III, and 3.4% and 3.2% for grade IV, respectively.

In our study, the percentage of ICRS grade I was higher in both groups than in Gooding's report and was significantly higher in C-ACI than in P-ACI. The difference between the first-generation ACI used by Gooding et al. and the three-dimensional culture ACI used in the present study may explain the difference in the results for the ICRS CRA.

Gooding et al.^6^ reported no significant difference between periosteal and collagenous membranes, even in first-generation ACI based on the modified Cincinnati score. Niemeyer et al.^24^ also compared the long-term results of the periosteum and collagen membrane in first-generation ACI using the Lysholm and IKDC scores. They found no significant difference in the 10-year follow-up survival rate between the two groups; however, the periosteum group had a significantly lower clinical score than the collagen membrane group. Barié et al.^23^ compared first-generation ACI using the periosteum and MACI in Medical Outcome Study Short-Form 36-Item Health Survey (SF-36) and reported no clinically significant differences between the two groups. The present study also showed no statistically significant difference in clinical scores between the two groups using three-dimensional cultured cartilage in ACI. Regardless of the type of covering, JACC® provided more stable clinical results than the first-generation ACI, suggesting that no difference was found in the clinical results between the two groups. Long-term follow-up is necessary in our cases, as there may be differences between the two groups.

In this study, the incidence of adverse events was reduced by using a collagen membrane instead of a periosteum. Previous reports^6,7,16,25^ have shown that using collagen membranes significantly reduces the incidence of adverse events. Adachi et al.^14^ reported the clinical results of ACI using JACC® with the periosteum and reported that 16% had graft hypertrophy that required shaving. Gooding et al.^6^ reported that adverse events were 36.4% and 0% in the periosteum and collagen membrane groups, respectively. Niemeyer et al.^16^ reported adverse event rates of 15.4% in the periosteum group and 1.9% in the collagen membrane group. Harris et al.^7^ reported that adverse events were 18% and 3% in the periosteum and collagen membrane groups, respectively. Gomoll et al.^25^ reported that 25.7% of patients required re-operation for graft hypertrophy within one year after P-ACI, whereas 5% of patients required similar re-operation after C-ACI. Previous studies^6,7,16,25^ have indicated that graft hypertrophy accounts for most adverse events. However, in the present study, hypertrophy was rarely observed as an adverse event. Surgical technique may have a significant impact on the occurrence of graft hypertrophy. To prevent graft hypertrophy, it is important to harvest the periosteum with as uniform a thickness as possible and suture the periosteum to the surrounding normal cartilage with moderate tension. In this study, all surgeries were performed by a single, experienced surgeon. This may explain why transplant hypertrophy was prevented in both the groups. Adverse events may also differ in multicenter studies that involve surgeons with varying degrees of experience.

Despite the development of periosteal substitutes, such as collagen membranes, some studies have shown the efficacy of the periosteum itself in chondrogenesis. O'Driscoll et al.^26^ investigated the effects of continuous passive motion on cartilage defects in rabbits implanted with only periosteum. They showed that continuous passive motion induced cartilage generation from the periosteum and that the repair tissue was primarily derived from chondroprogenitor cells present in the osteogenic layer of the periosteum. Grassel et al.^27^ co-cultured periosteum and chondrocytes to investigate the interaction between the periosteum and chondrocytes. They showed that the co-culture of periosteum with chondrocytes induced the expression of transforming growth factor-beta and collagen I, which may support the redifferentiation of the transplanted chondrocytes. However, Kajitani et al.^28^ showed that in ACI using JACC®, the periosteum has no humoral or cellular effects and that the periosteum is merely a cover, like a collagen membrane. With no significant difference in clinical outcomes, the use of periosteum would be cost-effective if the cost of collagen membranes was significantly increased or if the incidence of graft hypertrophy after periosteum-covered ACI was significantly reduced^29^. However, if the periosteum is not biologically superior, the current trend of replacing it with a collagen membrane is reasonable.

This study had some limitations. First, this was a retrospective cohort study. Retrospective comparisons by study design are susceptible to multiple biases (e.g., sample selection, lack of blinding, and differences in follow-up periods). Using collagen membranes instead of autologous periosteum has reduced operation time and invasiveness. Since 2019, collagen membranes have been used in all cases; hence, they have not affected case allocation. In addition, the patients had no choice regarding using the periosteum or collagen membrane; therefore, we believe that the lack of blinding would have little impact. However, it cannot be denied that the technical progress of the operator and the suitable patient selection gained through experience influences cases using collagen membranes. This may also explain why the number of tibial plateau cases, which were small in the first period, became even fewer in the second period. Many previous studies have been retrospective cohort studies for the same reason. Second, a biopsy was not performed during second-look arthroscopy, and histological comparison was not possible. A biopsy is an invasive and ethically challenging procedure. Therefore, magnetic resonance imaging has been shown to enable both structural and qualitative assessment^30^. In the future, we plan to compare both groups through structural and qualitative evaluations using magnetic resonance imaging. Third, the effect of concomitant surgical procedures on ACI outcomes was not considered in this study. In the future, a greater number of cases will allow us to consider the impact of each concomitant surgical procedure on the clinical outcomes of ACI.

## Conclusion

In ACI, using JACC®, a collagen membrane instead of the periosteum was effective. Although there was no significant difference in clinical outcomes between C-ACI and P-ACI, the superiority of using a collagen membrane was demonstrated in ICRS CRA during second-look arthroscopy and adverse event rate.

## Data Availability

Raw data were generated at Kameda Medical Center. Derived data supporting the findings of this study are available from the corresponding author [Y. K.] on request.
