# Author Correction: Comparative study on clinical outcomes in autologous chondrocyte implantation using three-dimensional cultured JACC^®^ with collagen versus periosteum coverings

**DOI:** 10.1038/s41598-024-70479-3

**Published:** 2024-08-22

**Authors:** Yuki Kato, Shin Yamada, Shuzo Takazawa, Soichi Hattori, Takuya Okada, Hiroshi Ohuchi

**Affiliations:** Department of Sports Medicine, Kameda Medical Center, 929 Higashi‑Cho, Kamogawa City, Chiba Prefecture 296‑8602 Japan

Correction to: *Scientific Reports* 10.1038/s41598-024-59798-7, published online 29 April 2024

The original version of this Article contained errors in Tables 1, where the values for “Other cartilage repairs combined with ACI”, “Microfracture” and “Osteochondral Autograft Transplantation” were misplaced.

Incorrect:

**Table:** 

Cartilage repair			
*Microfracture*	77% (53)	74% (25)	80% (28)
*Osteochondral Autograft*	29% (20)	18% (6)	40% (14)
*Transplantation*	72% (50)	68% (23)	77% (27)

Correct:

**Table:** 

Other cartilage repairs combined with ACI	77% (53)	74% (25)	80% (28)
*Microfracture*	29% (20)	18% (6)	40% (14)
*Osteochondral Autograft Transplantation*	72% (50)	68% (23)	77% (27)

The original Article has been corrected.

